# Tapia syndrome following anterior cervical surgery in a patient with diffuse idiopathic skeletal hyperostosis: a case report

**DOI:** 10.3389/fsurg.2025.1745141

**Published:** 2026-01-05

**Authors:** Liuxin Yan, Hongyang Gao, Zihao Zhen, Zhiming Yu

**Affiliations:** Department of Spine Surgery, The First Hospital of Hebei Medical University, Shijiazhuang, China

**Keywords:** airway management, anterior cervical surgery, cranial nerve injury, diffuse idiopathic skeletal hyperostosis (DISH), Tapia syndrome

## Abstract

**Background:**

Tapia syndrome is a rare complication characterized by concurrent injury to the hypoglossal and vagus nerves, most often associated with airway manipulation during general anesthesia. Patients with diffuse idiopathic skeletal hyperostosis (DISH) present unique anatomical challenges for anterior cervical surgery, increasing the risk of airway-related nerve injury.

**Case presentation:**

We report the case of a 73-year-old man with cervical DISH and myelopathy who underwent anterior cervical corpectomy and fusion (ACCF). Despite an uneventful intraoperative course, the patient developed postoperative hoarseness and delayed airway obstruction due to posterior tongue collapse. Laryngoscopy ruled out vocal cord injury. A diagnosis of Tapia syndrome was made based on the combination of hypoglossal and vagus nerve palsy. The patient required temporary tracheostomy and received glucocorticoid therapy, neurotrophic agents, and rehabilitation. All symptoms gradually resolved, and the patient was discharged after 40 days with near-complete recovery.

**Conclusion:**

This case emphasizes the need for careful airway management and early recognition of cranial nerve dysfunction in patients with DISH undergoing anterior cervical surgery. Individualized preoperative anatomical assessment, meticulous intraoperative airway control, and vigilant postoperative monitoring are essential to reduce the risk of this rare but significant complication.

## 1 Introduction

Cervical spondylosis is a common degenerative disorder of the cervical spine, and its clinical manifestations vary depending on the structures involved (1). The standard classification includes radiculopathic, myelopathic, vertebral artery, and sympathetic types, among which the radiculopathic form is most frequently encountered in clinical practice. Diffuse idiopathic skeletal hyperostosis (DISH) is a systemic, non-inflammatory disorder characterized by ossification of the anterior longitudinal ligament and entheses along the spine (2). When extensive osteophyte formation occurs in the anterior cervical region, it may lead to dysphagia, airway compression, and even increased difficulty in tracheal intubation (2, 3). For patients with significant symptoms, anterior cervical osteophytectomy and decompression can effectively relieve symptoms; however, due to the distorted anterior cervical anatomy and limited operative space, such procedures carry a potential risk of airway and neural complications. Tapia syndrome is a rare cranial neuropathy characterized by concurrent involvement of the hypoglossal nerve (cranial nerve XII) and the vagus nerve (cranial nerve X, particularly its recurrent laryngeal branch). Clinically, it presents with ipsilateral tongue deviation, dysarthria, and hoarseness (4). The most common etiology is airway manipulation under general anesthesia (5), especially in the context of prolonged endotracheal intubation, excessive cuff pressure, or extreme neck flexion or extension. Reported recovery times range from 3 to 22 months, with a median of approximately 9–12 months; complete recovery has been observed in about 30% of patients, incomplete recovery in roughly 39%, and no recovery in more than 26% of cases (6). Despite this generally guarded prognosis, the diagnosis is often delayed because early symptoms may be mild and easily mistaken for transient postoperative hoarseness or pharyngeal discomfort.

Here, we report a rare case of Tapia syndrome in a patient with DISH who developed postoperative hoarseness and delayed airway obstruction following anterior cervical corpectomy and fusion (ACCF). This case highlights the importance of individualized airway management and heightened vigilance for delayed cranial nerve dysfunction in patients with complex anterior cervical anatomy.

## 2 Case presentation

**Figure 1:**
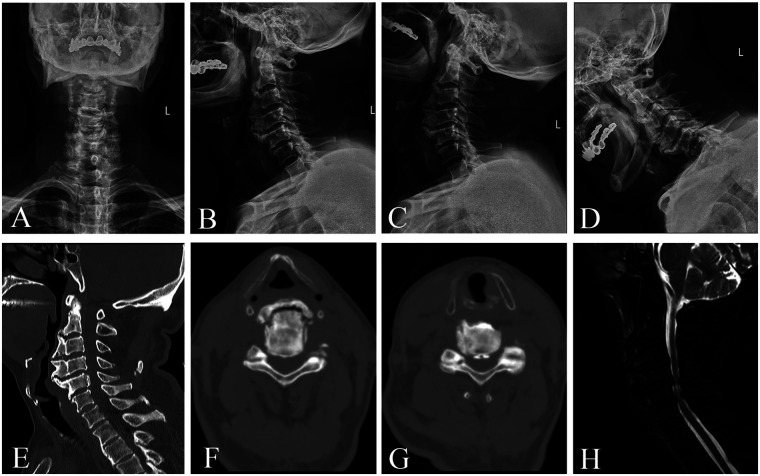
**(A,B)** cervical spine radiographs, AP and lateral views. **(C,D)** Cervical Flexion-Extension Radiographs. **(E–G)** Sagittal reformatted and axial CT images of the cervical spine at the C4 and C5 levels. **(H)** Sagittal T2-weighted magnetic resonance imaging (MRI) of the cervical spine.

A 73-year-old man, 170 cm in height and weighing 75 kg, presented with a two-year history of bilateral upper limb pain along the radial aspect, gait instability for one year, and dysphagia for one year. Two years earlier, he developed bilateral arm pain without any apparent precipitating factors. Conservative treatment provided little relief. One year prior to presentation, he began to experience unsteady gait described as a “walking on cotton” sensation, which was accompanied by progressive dysphagia. Neurological examination revealed no obvious sensory deficits over the body surface. Muscle strength of the bilateral deltoid, biceps, and triceps was decreased to grade IV. The triceps tendon reflexes were hyperactive bilaterally, while the biceps and brachioradialis reflexes were absent. Knee jerk reflexes were brisk on both sides. Bilateral Babinski and Hoffmann signs were positive. Cervical x-rays (Figures 1A–D) demonstrated large anterior osteophytes at the C3 and C4 vertebral levels, with smaller osteophytes at adjacent segments. Computed tomography (CT) of the cervical spine (Figures 1E–G) showed ossification of the posterior longitudinal ligament at the posterior margin of the C5 vertebral body. Magnetic resonance imaging (MRI) of the cervical spine (Figure 1H) revealed disc protrusions at C3/4, C4/5, C5/6, and C6/7, with spinal canal stenosis and possible spinal cord myelomalacia at the C4/5 level. Representative imaging findings are shown in Figure 1. Based on the above clinical and radiological findings, the patient was scheduled to undergo ACCF with resection of the anterior osteophytes at C4 and C5.

## 3 Surgical procedure

The patient was placed in the supine position. Anesthesia was induced with intravenous propofol and remifentanil, and neuromuscular blockade was achieved using rocuronium. Nasotracheal intubation was performed under indirect visualization using a fiberoptic bronchoscope with a 7.0-mm reinforced endotracheal tube. The tube was advanced to a depth of 27 cm at the level of the upper incisors. After successful intubation, the neck was mildly extended with a support pad placed beneath the shoulders, and the head was secured in a neutral position. After standard sterile preparation and draping, a right-sided oblique incision approximately 8 cm in length was made along the medial border of the sternocleidomastoid muscle. The skin, subcutaneous tissue, and platysma were divided layer by layer. The surgical approach was developed medial to the carotid sheath, retracting the trachea and esophagus to the left to expose the anterior surface of the cervical spine. The hypertrophic anterior osteophytes were identified and resected using rongeurs and a high-speed burr. Under C-arm fluoroscopic guidance, the C5 vertebral body was localized. The anterior longitudinal ligament was incised, and the intervertebral discs at C4–C5 and C5–C6 were removed with curettes to expose the spinal canal. Intraoperative exploration revealed posterior protrusion of disc material at both levels, compressing the dura mater. The herniated disc tissue was completely excised, and a subtotal corpectomy of C5 was performed to achieve thorough decompression. After decompression, dural expansion was clearly observed. An appropriately sized titanium cage filled with autologous bone granules was inserted between C4 and C6 to restore spinal alignment. A titanium anterior cervical plate was applied to the vertebral bodies, and screws of suitable length were inserted after precise drilling. Fluoroscopy confirmed satisfactory alignment and fixation. After meticulous hemostasis and instrument count verification, a negative-pressure drainage tube was placed. The wound was closed in layers with absorbable sutures for the deep fascia and subcutaneous tissues, followed by skin closure and sterile dressing application. The total operative time was approximately 5 h, with an estimated blood loss of 150 ml and intraoperative autologous blood reinfusion of 244 ml. After anesthesia recovery, the patient was transferred to the ward with a cervical collar in place for stabilization.

## 4 Postoperative course

**Figure 2:**
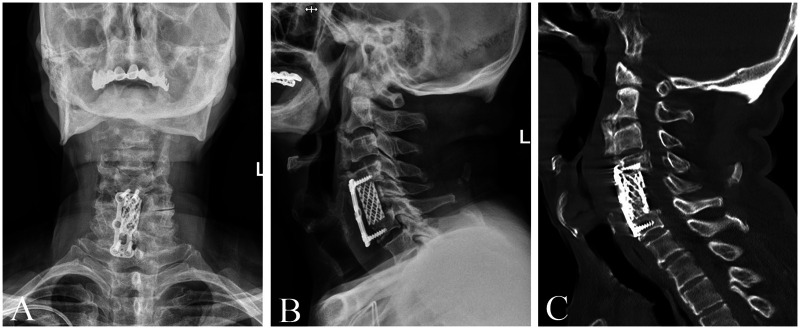
**(A,B)** cervical spine radiographs (AP and lateral views) at 2-month follow-up. **(C)** Postoperative follow-up cervical spine CT.

After surgery, the patient was transferred to the post-anesthesia care unit (PACU) for monitoring. Upon emergence from anesthesia, consciousness was fully regained, the oxygen saturation remained above 95%, and muscle strength of all skeletal muscle groups had largely recovered. After anesthesiologist assessment, the patient was transferred back to the general ward. In the ward, his blood pressure was stable at 120/70 mmHg, and his respiratory rhythm was regular. Except for increased sputum production and hoarseness, his general condition remained stable. On postoperative day (POD) 1, the patient's preoperative symptoms had significantly improved, though persistent hoarseness was noted. On POD 2, the patient suddenly developed agitation, dyspnea, and expiratory wheezing in the early morning. The wound drainage was unremarkable, with outputs of 40 ml, 20 ml, and 5 ml on POD 1, POD 2, and POD 3, respectively, without any sudden increase or evidence of fresh bleeding. There was no neck swelling, wound tension, or new focal neurological deficit at the time of deterioration. Nebulized salbutamol and budesonide were administered, but symptoms did not improve significantly. An urgent consultation with the intensive care unit (ICU) team was obtained. The intensivist recommended intravenous injection of methylprednisolone sodium succinate (80 mg dissolved in 5 ml of normal saline) and close observation of respiratory response. When the tongue was brought forward, the dyspnea was markedly relieved, suggesting airway obstruction secondary to posterior tongue collapse. On protrusion, the tongue deviated to the right, consistent with right hypoglossal nerve palsy. A psychiatric consultation was requested, and delirium was considered as a possible contributing factor. Once the patient's condition stabilized, an emergency cranial CT scan was performed, which revealed no evidence of new cerebral infarction. Otolaryngology consultation and laryngoscopic examination showed no vocal cord or glottic injury. After discussion with the family, the patient was transferred to the intensive care unit for further management. During his ICU stay, he experienced recurrent episodes of airway obstruction due to posterior tongue collapse, and a tracheostomy was performed to secure the airway. Following stabilization in the ICU, the patient was returned to the general ward to continue treatment. Postoperatively, he received glucocorticoids, neurotrophic agents, and rehabilitation therapy. After 40 days, the tracheostomy tube was successfully removed, and both hoarseness and tongue collapse gradually resolved. The patient was subsequently discharged in stable condition. A detailed timeline of postoperative symptoms, interventions, and outcomes is presented in Table 1. This chronological summary provides a concise overview of the patient's clinical evolution from surgery to discharge. Follow-up cervical x-rays (Figures 2A,B) and CT images (Figure 2C) showed successful resection of the anterior osteophytes and the ossified posterior longitudinal ligament at C5.

**Table 1:** Postoperative clinical course of the patient.

Postoperative day (POD)	Clinical findings/Symptoms	Interventions and management	Outcome/Response
POD 0 (day of surgery)	Consciousness fully regained after anesthesia; oxygen saturation >95%; muscle strength recovered; hoarseness noted; stable vital signs.	Routine monitoring in PACU; transferred to general ward after stabilization.	Stable condition.
POD 1	Preoperative symptoms markedly improved; persistent hoarseness present; normal respiration.	Symptomatic observation and vocal rest.	Hoarseness unchanged.
POD 2 (early morning)	Sudden agitation, dyspnea, expiratory wheezing.	Nebulized salbutamol and budesonide; ICU consultation; IV methylprednisolone 80 mg; psychiatric evaluation.	Dyspnea relieved when the tongue was brought forward; airway obstruction due to tongue collapse suspected.
POD 2 (later)	Persistent intermittent airway obstruction; no cerebral infarction on CT; normal laryngoscopy.	Transferred to ICU; tracheostomy performed to secure airway.	Airway stabilized post-tracheostomy.
POD 3–40	ICU and ward recovery phase; received glucocorticoids, neurotrophic agents, and rehabilitation therapy.	Gradual improvement in tongue mobility and voice.	Tracheostomy decannulated after 40 days; hoarseness and tongue collapse resolved; discharged in stable condition.

POD, postoperative day; PACU, post-anesthesia care unit; ICU, intensive care unit.

## 5 Discussion

Preoperative lateral cervical radiographs revealed extensive anterior osteophyte formation extending from C2 to C5, with additional calcific proliferations along the anterior margins of the remaining vertebral bodies. These imaging findings met the diagnostic criteria for DISH (7). Based on these features, the patient was diagnosed with DISH. The extensive anterior osteophytes had resulted in progressive dysphagia, and when combined with the patient's cervical spondylotic myelopathy, surgical intervention was indicated. Postoperatively, the patient developed hoarseness and delayed posterior tongue collapse accompanied by delirium, which ultimately required tracheostomy and gradually resolved with appropriate management. This case underscores two key points of clinical significance. First, the anterior cervical osteophytes associated with DISH may substantially alter the anatomical course or spatial relationship of the hypoglossal and vagus nerves, thereby increasing the risk of mechanical traction or compression injury during tracheal intubation or intraoperative retraction. Second, prolonged surgery and postoperative delirium may exacerbate or unmask subclinical peripheral nerve injury, leading to the emergence of clinically evident deficits under systemic stress—a phenomenon that may be described as the conversion of subclinical injury into overt neurological dysfunction.

From a differential-diagnostic standpoint, an acute postoperative cervical hematoma must be considered first when a patient develops sudden airway compromise on POD 2. In our case, however, several clinical features argued against a compressive hematoma as the primary cause of deterioration. First, there was no progressive neck swelling, wound tension, or new limb weakness. Second, the closed-suction drain output decreased steadily (40 ml, 20 ml, and 5 ml on POD 1–3, respectively) without any abrupt increase or change in character to suggest active bleeding. Third, the patient's dyspnea improved immediately when the tongue was brought forward, which is more consistent with dynamic upper-airway obstruction due to posterior tongue collapse than with a fixed external mass effect. Taken together, these findings made a significant postoperative hematoma unlikely and supported late-onset cranial neuropathy, unmasking a previously subclinical injury of the hypoglossal and vagus nerves under systemic stress, as the most plausible explanation for the clinical course. A postoperative cervical CT or MRI was not obtained at the time of symptom onset, which is a limitation of this report; however, the clinical picture and drainage profile made a clinically relevant hematoma unlikely.

Both airway manipulation and intraoperative surgical retraction may contribute to hypoglossal and vagus nerve injury in this setting. In the present case, the distorted anterior cervical anatomy required leftward retraction of the trachea and esophagus during a right-sided anterior approach, increasing tension on adjacent neurovascular structures. At the same time, the curvature of the reinforced endotracheal tube may have caused lateral compression against the oropharyngeal and hypopharyngeal walls, thereby compressing the nearby nerves. The delayed onset of severe symptoms on POD 2 suggests that an initially mild, subclinical neuropathy may have been exacerbated by postoperative delirium, systemic stress, or positional changes, eventually manifesting as clinically overt Tapia syndrome. No intraoperative thyroid injury was observed, and the patient exhibited no clinical signs suggestive of hypocalcemia, such as perioral numbness or carpopedal spasm, making these etiologies unlikely.

The patient's body mass index (BMI) was 25.95, falling within the overweight range. To minimize the risk of compression injury to the laryngeal wall and surrounding tissues from the endotracheal tube or cuff, a smaller-sized reinforced endotracheal tube (7.0 mm) was selected. Intubation was successfully achieved on the first attempt under indirect visualization using a fiberoptic bronchoscope. The tube was secured at both ends—with adhesive tape at the mouth and an inflated cuff within the trachea—to maintain a stable airway. Despite these precautions, the patient developed postoperative hoarseness, which, in combination with subsequent tongue muscle paralysis, was consistent with the typical presentation of Tapia syndrome. This suggests that even the use of a smaller-diameter endotracheal tube does not fully eliminate the risk of Tapia syndrome. The most plausible explanation is that inexperienced intraoperative retraction may have caused the curved mid-portion of the endotracheal tube to deviate laterally from the midline, exerting pressure on the lateral walls of the oropharynx and hypopharynx and thereby compressing the adjacent vagus and hypoglossal nerves. It is noteworthy that postoperative hoarseness is relatively common among patients undergoing general anesthesia with endotracheal intubation. The incidence of hoarseness on the day of surgery has been reported to reach up to 49%, though it typically decreases rapidly over time (8). In clinical practice, postoperative hoarseness is often underestimated or overlooked; however, in rare cases, it may serve as an early warning sign of potential cranial nerve injury such as Tapia syndrome.

Previous studies have reported that a reduced distance between the mandibular angle and the anterior margin of the cervical vertebral body on neutral lateral cervical radiographs may represent a risk factor for postoperative Tapia syndrome in patients undergoing posterior cervical surgery under general anesthesia with oral endotracheal intubation (9). Although the present patient underwent anterior cervical surgery, the presence of massive anterior osteophytes was noteworthy. Lateral cervical x-rays and CT scans demonstrated that the line connecting both mandibular angles intersected the osteophytic mass, suggesting a narrowed anatomical corridor for the course of the vagus and hypoglossal nerves. During surgery, the patient's neck was positioned in mild hyperextension, which may have slightly expanded the neural pathway space. However, preoperative lateral radiographs obtained in the extended position revealed that even in hyperextension, the potential space for these nerves remained narrower than in normal individuals. Therefore, comprehensive preoperative planning tailored to the patient's specific anatomy is essential. Such individualized assessment not only helps minimize perioperative complications and improve postoperative satisfaction, but also reflects the core principles of precision and personalized surgical management in modern spine surgery (10).

The total operative time exceeded five hours, which was notably longer than that of a typical ACCF. The main reason was the patient's preoperative dysphagia, necessitating surgical decompression of the anterior cervical osteophytes that had failed to respond to conservative therapy. In elderly male patients, prolonged surgery constitutes a significant stress-related risk factor (11). In this case, the patient developed delirium with severe tongue muscle paralysis on postoperative day two, which was likely the result of multiple synergistic factors. First, the abnormal preoperative cervical anatomy, combined with intraoperative retraction by the assistant, may have caused mechanical injury to the recurrent laryngeal and hypoglossal nerves. The initial degree of nerve injury may have been compensated by neural reserve, thereby masking corresponding clinical manifestations in the early phase. However, the prolonged surgical duration and the onset of postoperative delirium likely impaired central neural control of the peripheral nerves, serving as the trigger for the manifestation of severe postoperative neurological deficits. Accumulating multidisciplinary evidence suggests that many forms of neural injury may remain subclinical or latent under resting conditions. When exposed to external physiological, psychological, or mechanical stressors—such as hypoxia, sleep deprivation, emotional stress, or positional/mechanical changes—these latent injuries may become clinically overt, presenting with functional deficits or radiological activation (12–14). In short, this case exemplifies the conversion of subclinical nerve injury into clinically manifest dysfunction under systemic stress. Based on the above analysis, several preventive measures are proposed: (a) Preoperative evaluation of the mandibular angle–anterior vertebral margin distance and airway anatomy, with three-dimensional CT reconstruction when necessary. (b) During endotracheal intubation, select the smallest suitable internal diameter tube, monitor cuff volume and pressure, and use low-pressure cuffs. (c) Avoid excessive neck hyperextension or rotation, and periodically verify endotracheal tube position intraoperatively. (d) Minimize the duration of airway retraction and schedule intermittent relaxation periods during surgery. Postoperatively, any occurrence of hoarseness or dysphagia should prompt early laryngoscopic and neurological evaluation, followed by early rehabilitation intervention if indicated. For high-risk patients, preoperative consultation with the anesthesiology team is essential to determine the optimal intubation approach and assess the need for intraoperative airway pressure monitoring.

Most cases of Tapia syndrome represent a transient neuropathy, and once the diagnosis is established, early intervention with glucocorticoids, neurotrophic agents, and functional rehabilitation can significantly improve outcomes. A formal swallow study was not performed in this case; however, the diagnosis was established based on the characteristic combination of hypoglossal and vagus nerve deficits, normal laryngoscopic findings, and the absence of central lesions on cranial CT. With timely treatment and early rehabilitation involvement, neurological recovery is typically achieved within several weeks to months (4, 6, 15). In this case, the patient achieved near-complete recovery of all symptoms and was discharged 40 days postoperatively. Neuromodulation techniques, such as hypoglossal nerve stimulation, have been explored primarily in obstructive sleep apnea, but evidence supporting their use in Tapia syndrome is lacking. At present, conservative management with corticosteroids, neurotrophic therapy, and rehabilitation remains the standard of care.

Through this case report, we aim to raise clinical awareness of Tapia syndrome among spine surgeons. Special attention should be paid to patients with diffuse idiopathic skeletal hyperostosis (DISH) who present with preoperative cervical dysphagia, as they may be at higher risk for nerve compression and positioning-related injury during surgery. Careful intraoperative manipulation and meticulous postoperative monitoring of voice and swallowing function are essential to ensure early recognition and management of this rare but potentially distressing complication.

## 6 Conclusion

In patients with diffuse idiopathic skeletal hyperostosis (DISH) undergoing anterior cervical surgery, airway-related nerve injury should be recognized as a significant intraoperative and postoperative complication. Individualized preoperative anatomical assessment, meticulous intraoperative airway management, and heightened postoperative vigilance are essential to minimize the risk of severe complications and to enable timely intervention when they occur.

## Data Availability

The original contributions presented in the study are included in the article/Supplementary Material, further inquiries can be directed to the corresponding author.
